# Mistletoe-induced carbon, water and nutrient imbalances are imprinted on tree rings

**DOI:** 10.1093/treephys/tpae106

**Published:** 2024-08-20

**Authors:** Ester González de Andrés, Antonio Gazol, José Ignacio Querejeta, Michele Colangelo, J Julio Camarero

**Affiliations:** Conservación de Ecosistemas, Instituto Pirenaico de Ecología (IPE-CSIC), Avda Montañana 1005, 50059 Zaragoza, Spain; Conservación de Ecosistemas, Instituto Pirenaico de Ecología (IPE-CSIC), Avda Montañana 1005, 50059 Zaragoza, Spain; Centro de Edafología y Biología Aplicada del Segura (CEBAS-CSIC), Campus de Espinardo, 30100 Murcia, Spain; Conservación de Ecosistemas, Instituto Pirenaico de Ecología (IPE-CSIC), Avda Montañana 1005, 50059 Zaragoza, Spain; Scuola di Scienze Agrarie, Forestali, Alimentari e Ambientali, Università della Basilicata, Viale dell'Ateneo Lucano 10, 85100 Potenza, Italy; Conservación de Ecosistemas, Instituto Pirenaico de Ecología (IPE-CSIC), Avda Montañana 1005, 50059 Zaragoza, Spain

**Keywords:** intrinsic water-use efficiency, Scots pine, silver fir, stochiometric ratio, stomatal conductance, *Viscum album*

## Abstract

Mistletoes are xylem-tapping hemiparasites that rely on their hosts for water and nutrient uptake. Thus, they impair tree performance in the face of environmental stress via altering the carbon and water relations and nutritional status of trees. To improve our understanding of physiological responses to mistletoe and ongoing climate change, we investigated radial growth, stable carbon and oxygen isotopic signals, and elemental composition of tree rings in silver fir (*Abies alba* Mill.) and Scots pine (*Pinus sylvestris* L.) forests infested with *Viscum album* L. We compared temporal series (1990–2020) of basal area increment (BAI), intrinsic water-use efficiency (iWUE), oxygen isotope composition (δ^18^O), nutrient concentrations and stoichiometric ratios between non-infested (NI) and severely infested (SI) fir and pine trees from populations located close to the xeric distribution limit of the species in north-eastern Spain. The SI trees showed historically higher growth, but the BAI trend was negative for more than three decades before 2020 and their growth rates became significantly lower than those of NI trees by the mid-2010s. Mistletoe infestation was related to an enhanced sensitivity of radial growth to vapour pressure deficit (atmospheric drought). The SI trees showed less pronounced iWUE increases (fir) and lower iWUE values (pine) than NI trees. The lower tree-ring δ^18^O values of SI trees may be the result of several superimposed effects operating simultaneously, including leaf-level evaporative enrichment, source water isotopic signals, and anatomical and phenological differences. We observed a deterioration of potassium (K) nutrition in tree-ring wood of both species in SI trees, along with accumulation of manganese (Mn). We suggest that such nutritional patterns are driven by the indirect effect of mistletoe-induced drought stress, particularly in pine. The combined analyses of different physiological indicators imprinted on tree rings provided evidence of the progressive onset of carbon, water and nutrient imbalances in mistletoe-infested conifers inhabiting seasonally dry regions.

## Introduction

Mistletoes are the most successful group of hemiparasitic plants, capable of colonizing aerial parts of many tree species in ecosystems worldwide (Glatzel and Geils 2009). Mistletoes are a diverse group that encompasses nearly 1600 species distributed among five families in the Santalales order (Muche et al. 2022). They play important roles at the ecosystem scale, such as enhancing nutrient cycling and promoting biodiversity (Griebel et al. 2017). Mistletoes and their hosts can co-exist for years without major impacts on trees in the absence of resource limitations (Zuber 2004). However, mistletoes can also exacerbate the effect of environmental stresses such as drought (Rigling et al. 2010), thereby emerging as important contributing factor to canopy dieback and tree mortality (Dobbertin and Rigling 2006). Climatic projections forecast increases in atmospheric water demand, combined in many regions with reductions in soil water availability (Zhou et al. 2019; IPCC 2021), whereby climate change-related forest disturbances are expected to increase significantly (Brodribb et al. 2020). Concurrently, the distribution range of some mistletoe species is expected to expand (Sangüesa-Barreda et al. 2018; Walas et al. 2022), so mistletoe-induced damage in increasingly drought-stressed forests is likely to intensify. In Europe, *Viscum album* L. (Viscaceae) is the most widely distributed mistletoe species (Zuber 2004), which poses a major threat to coniferous forests due to its significant and rapid expansion (Dobbertin and Rigling 2006; Walas et al. 2022). Understanding how trees respond to the simultaneous impacts of mistletoe infestation and ongoing climate change is therefore essential for improving model projections of forest performance and designing suitable management strategies.

Mistletoes tap into the host xylem using a perennial endophytic system called haustorium (Glatzel and Geils 2009). The lower water potential of mistletoes compared with host leaves allows them to maintain high stomatal conductance and transpiration rates to achieve a continuous water uptake from the xylem of trees (Ehleringer et al. 1985). This results in hydraulic dysfunction and enhanced drought sensitivity of mistletoe-infested trees, as indicated by decreases in stomatal conductance (Zweifel et al. 2012) or loss of hydraulic conductivity (Reblin and Logan 2015; Griebel et al. 2022). Furthermore, reductions in nonstructural carbohydrate concentration (Sangüesa-Barreda et al. 2012; Yan et al. 2016), leaf size and crown leaf area (Meinzer et al. 2004; Ozturk et al. 2022), intrinsic water-use efficiency (iWUE; Sangüesa-Barreda et al. 2013) and ultimately radial growth decline (Rigling et al. 2010; Kollas et al. 2018) provide evidence for mistletoe-induced impairment of carbon assimilation. In addition, there is a debate about the potential heterotrophic behaviour of mistletoes, i.e. obtaining carbon assimilates via acropetal transport from the host xylem to the mistletoe via the transpiration stream since no phloem connection is established between hemiparasite and host (Escher et al. 2004; Glatzel and Geils 2009; Scalon and Wright 2015).

As for mistletoe nutrition, the sap uptake and transfer from the host xylem via haustorium is the passive mechanism by which these hemiparasites absorb nutrients (Muche et al. 2022). This causes the accumulation of nutrients in mistletoe tissues compared with their hosts (Scalon et al. 2013; Wang et al. 2023). Previous research has reported decreases in nutrient contents (Yan et al. 2016; Mutlu et al. 2016*b*) or alteration of metabolic processes (Mutlu et al. 2016*a*; Lázaro-González et al. 2021) in trees subjected to severe mistletoe infestation. Adequate levels of macronutrients (e.g. nitrogen [N], phosphorus [P], potassium [K], calcium [Ca], sulphur [S] and magnesium [Mg]) and micronutrients (e.g. iron [Fe], copper [Cu] and manganese [Mn]) are required for critical physiological processes such as photosynthesis or hydraulic function, which in turn determine mechanisms of drought stress avoidance of plants (da Silva et al. 2011). Therefore, mistletoe-induced nutrient impairment can further aggravate the disruption of the carbon-water balance of infested trees (McDowell 2011; Gessler et al. 2017).

 Growth patterns, along with the physiological processes that drive them, in mistletoe-infested trees can be reconstructed with the information imprinted in tree rings. Firstly, tree-ring width (TRW) is highly sensitive to environmental conditions (Babst et al. 2019) and specific radial growth patterns have been related to impending tree mortality including long-term growth rates, high synchronicity or low growth resilience (Cailleret et al. 2017; DeSoto et al. 2020). Secondly, the carbon (C) and oxygen (O) stable isotope composition of tree rings provides interesting clues to the carbon and water relationships of trees at the time the ring was formed (McCarroll and Loader 2004). The carbon isotopic composition of plant C assimilates is mainly determined by the interactive responses of photosynthetic rates and stomatal conductance to environmental conditions (Cernusak et al. 2013). Meanwhile, the oxygen isotopic composition of plant tissues mainly reflects the source water isotopic signature and is further affected by evaporative effects at leaf level during photosynthesis (Barbour 2007; Gessler et al. 2014; Wang et al. 2021). The ‘so called’ dual-isotope approach has been applied to make inferences regarding the responses of photosynthetic capacity (*A*) and stomatal conductance (*g_s_*) to environmental variables (Scheidegger et al. 2000; Siegwolf et al. 2023) although further considerations regarding postphotosynthetic isotopic fractionation effects are needed when interpreting stables isotopes in tree rings (Roden and Siegwolf 2013).

In addition, nutrient concentration patterns in wood have been recognized as a valuable source of information on environmental changes and tree nutritional status (e.g. Kuang et al. 2008; Smith et al. 2014; Hevia et al. 2018; González de Andrés et al. 2021) and can be used as indicator of basic physiological functions (da Silva et al. 2011; Fromm 2010). The nutrient content of the xylem undergoes ontogenetic changes, so that a decrease in cation concentrations from pith to cambium driven by nutrient translocation to more metabolically active tissues has been commonly described (Helmisaari and Siltala 1989; Smith et al. 2014; Hevia et al. 2019). Cation concentrations in sapwood can also dynamically increase in response to environmental stress to enhance hydraulic conductivity (Nardini et al. 2011). Despite such internal translocation of nutrients in wood, the assessment of nutrient concentrations and stoichiometric ratios in tree woody tissues has permitted the identification of nutritional deterioration trends associated with drought-induced canopy dieback and tree mortality (Hevia et al. 2019; González de Andrés et al. 2021, 2022). A recent meta-analysis has identified common patterns including lower K, P, Fe and Cu and higher Mn concentrations in trees affected by drought-induced mortality (He et al. 2024). Moreover, stoichiometric imbalances have been associated with drought stress, such as increases in N:P and N:K ratios that indicate P and K limitation of growth (Güsewell 2004; Salazar-Tortosa et al. 2018). Evaluation of the elemental concentrations and ratios of tree rings can further improve our understanding of the impact of mistletoe on tree performance, given the importance of adequate nutrition in response to environmental stress (Gessler et al. 2017). However, the combined analysis of these different indicators from tree rings (radial growth, C and O isotopic signature, and nutrient composition) has not been previously addressed to retrospectively assess tree responses to mistletoe infestation under increasing climatic stress.

In this study, we investigated the impacts of *V. album* on tree growth, C and O isotopic signals, and elemental composition in tree rings reflecting time-integrated photosynthetic gas exchange and nutrition over time on two major European conifers, Scots pine (*Pinus sylvestris* L.) and silver fir (*Abies alba* Mill.). These species reach their southern and xeric distribution limit in the Iberian Peninsula (Caudullo et al. 2017), where episodes of drought-induced tree mortality have been reported during the last decades (e.g. Galiano et al. 2010; Camarero et al. 2011). Our sampled forests are located in seasonally dry regions of north-eastern Spain, thus facing the dual stress imposed by drought and mistletoe infestation (Bose et al. 2024). We compared TRW, C and O stable isotope composition, and elemental concentrations in tree-ring wood of co-occurring non-infested (NI) and severely infested (SI) trees of both conifers and related them to climate variables over the last three decades. We tested the specific hypotheses that (i) radial growth of mistletoe-infested trees would rapidly decline and be more negatively impacted by drought stress; (ii) mistletoe infestation would impair carbon assimilation and reduce *g_s_*, thereby affecting tree-ring C and O isotopic signals; and (iii) nutrient concentration in tree rings would be lower and would show more negative temporal trends combined with nutrient imbalance in mistletoe-infested trees compared with their healthy counterparts as a consequence of the nutrient capture by the hemiparasite.

## Materials and methods

### Study species and study sites

Silver fir and Scots pine are two major tree species in Europe with social, economic and ecological relevance due to timber production and the provision of ecosystem services such as soil protection, water regulation, or recreational uses (del Río et al. 2017; Vitasse et al. 2019). They are the main hosts of *Viscum album* subsp. *abietis* (Wiesb.) Abrom. and *Viscum album* subsp. *austriacum* (Wiesb.) Vollm., respectively; the most widespread mistletoe species colonizing European conifer forests (Zuber 2004). These taxa are very similar morphologically and can only be distinguished by molecular methods (Zuber and Widmer 2009), so hereafter they will be referred to simply as *V. album*.

We studied two conifer forests which showed a widespread presence of mistletoe, including some trees that were SI and presented signs of canopy dieback. They were a silver fir stand in the Pyrenees (Villanúa site) and a Scots pine stand in the Iberian System (Moscardón site) (Table 1). Companion tree species at the Villanúa silver fir site are *Pinus sylvestris* L. and *Fagus sylvatica* L., and the understory is dominated by *Buxus sempervirens* L., *Ilex aquifolium* L. and *Crataegus monogyna* Jacq. Common understory species at Moscardón Scots pine site are *Quercus faginea* Lam., *Quercus ilex* L., *Pinus nigra* J.F. Arn., *Acer monspessulanum* L., *Amelanchier ovalis* Medik, *C. monogyna* and *Genista Scorpius* (L.) DC. Climate at the Villanúa site is continental with relatively cool and wet summers, whereas climate at the Moscardón site is Mediterranean-continental with a marked period of summer drought (mainly July and August, Fig. S1a available as Supplementary data at *Tree Physiology* Online). The coldest and warmest months at both sites are January and July to August, respectively. Annual precipitation is almost twice as high in Villanúa (954 mm) as in Moscardón (550 mm), with the driest months being July to August in both sites (Table 1, Fig. S1a available as Supplementary data at *Tree Physiology* Online).

**Table 1:** Characteristics (mean ± standard error) of sampled sites and trees for NI and SI trees by mistletoe. Different letters indicate significant (*P* < 0.05) differences between infestation classes according to one-way ANOVA tests.

	***Abies alba***		***Pinus sylvestris***	
	**NI trees**	**SI trees**	**NI trees**	**SI trees**
Site (province)	Villanúa (Huesca)		Moscardón (Teruel)	
Latitude N	42° 40′ 42′′		40° 18′ 40′′	
Longitude W	0° 30′ 08′′		1° 32′ 53′′	
Elevation (m a.s.l.)	1310		1440	
Aspect	NW		E – W	
MAT (°C)^1^	9.59 ± 0.11		8.75 ± 0.11	
MAP (mm)^1^	954 ± 24		551 ± 15	
DBH (cm)	35.4 ± 1.3a	38.8 ± 2.2a	34.5 ± 1.8a	39.4 ± 1.6b
Tree height (m)	20.0 ± 0.7a	21.3 ± 0.6a	15.7 ± 0.5a	15.5 ± 0.6a
Tree age at 1.3 m (yr)	118 ± 7a	117 ± 5a	90 ± 5a	106 ± 4b
Crown defoliation (%)	11.9 ± 4.1a	64.3 ± 4.3b	20.5 ± 2.4a	74.5 ± 2.1b
No. sampled trees (No. radii)	16 (24)	15 (18)	38 (72)	21 (39)
TRW 1950–1989	0.88 ± 0.11a	1.29 ± 0.18b	1.52 ± 0.08a	1.57 ± 0.15a
TRW 1990–2020	1.23 ± 0.14b	0.89 ± 0.11a	1.08 ± 0.07b	0.68 ± 0.07a
AC^2^	0.83 ± 0.02a	0.79 ± 0.03a	0.68 ± 0.02a	0.81 ± 0.02b
EPS^2^	0.874	0.856	0.972	0.955
*Rbar* ^2^	0.315	0.271	0.477	0.500
MS^2^	0.165	0.176	0.229	0.237

Variables’ abbreviations: mean annual temperature (MAT), mean annual precipitation (MAP), mean inter-series correlation (*Rbar*).

^1^Calculated for the period 1960–2021 based on 0.1° gridded E- OBS v. 27.0e database (Cornes et al. 2018).

^2^Calculated for the period 1950–2020 on raw (AC) or standardized (*Rbar*, MS, EPS) ring-width values.

### Climate data

The Standardized Precipitation Evapotranspiration Index (SPEI) was used to describe drought intensity and used as a proxy of soil moisture (Vicente-Serrano et al. 2010). This is a normalized drought index based on the cumulative difference between precipitation and potential evapotranspiration, which can be calculated at different temporal resolutions. Drought index data for each study site were extracted from the 1.1-km^2^ gridded series of the Spanish SPEI database (Vicente-Serrano et al. 2017). Vapour pressure deficit (VPD) was used as a measure of evaporative water demand. The VPD was estimated as the difference between saturation vapour pressure (SVP) and actual vapour pressure (AVP) (Williams et al. 2013). We downloaded temperature and AVP data from the 0.5° gridded CRU database v. 4.07 (Harris et al. 2020). SVP was calculated following (Williams et al. 2013):


(1) \begin{equation*} \mathrm{SVP}={a}_0+T\left({a}_1+T\left({a}_2+T\left({a}_3+T\left({a}_4+T\left({a}_5T{a}_6\right)\right)\right)\right)\right), \end{equation*}

where *T* is air temperature in degrees Celsius, *a_0_* = 6.1078, *a_1_* = 4.4365 × 10−1, *a_2_* = 1.4289 × 10−2, *a_3_* = 2.6506 × 10−4, *a_4_* = 3.0312 × 10−6, *a_5_* = 2.0341 × 10−8, and *a_6_* = 6.1368 × 10−11. The VPD data were normalized to have a mean of zero and standard deviation of one at each site. In order to characterize tree response to water deficit, we obtained summer (June to August) VPD and SPEI series, which is the season in which the tree species showed the strongest radial growth–climate relationship at the study sites (Camarero et al. 2015; Gazol et al. 2023).

Monthly δ^18^O of precipitation was retrieved from the Global Network of Isotopes in Precipitation (IAEA/WMO 2021). We selected the closest station to each study site: Noguera de Albarracín (40° 27′ 29″ N, 1° 35′ 55″ W, 1449 m a.s.l.; period: 2013–2015) and Puerto Orihuela (40° 30′ 25″ N, 1° 39′ 05″ W, 1720 m a.s.l.; period: 2016–2021) for the Moscardón site, and Sallent de Gállego—La Sarra (42° 46′ 22″ N, 0° 19′ 53″ W, 1285 m a.s.l.; period: 2013–2021) for the Villanúa site. To obtain time series of δ^18^O of precipitation spanning the same period as δ^18^O of tree rings, we interpolated data with those of the Madrid—El Retiro station (40° 24′ 43″ N, 3° 40′ 41″ W, 667 m a.s.l.; period: 1986–2021) using linear regressions.

### Field sampling

In total, 90 dominant and codominant trees were selected for the present study (31 fir trees and 59 pine trees). At each forest stand, half of the trees were noninfested or slightly infested (NI trees), and the other half were SI trees by mistletoe. The mistletoe infestation degree was estimated using a modified version of the Hawksworth scale (Hawksworth 1977). Tree crown was divided into three similar vertical parts and each third was scored with 0 (absence of mistletoe), 1 (moderate presence of mistletoe) or 2 (high presence of mistletoe), and finally, the contribution of each third was summed to obtain a tree-level value (Sangüesa-Barreda et al. 2012). The NI trees were considered those with infestation values under 2, while SI trees were those equal to or greater to 4. We also assessed crown defoliation by visual assessment of crown transparency as an estimate of tree vitality (Dobbertin 2005). For each selected tree, we measured diameter at breast height (DBH) and tree height using tapes and a laser range finder (Nikon Forestry Pro II), respectively. Sampling was carried out in autumn 2021 in the fir stand and summer 2020 in the pine stand. For the sake of simplicity, the end of the study period for both species will be named 2020, although the last ring for firs is 2021 and 2019 for pines.

**Figure 1:**
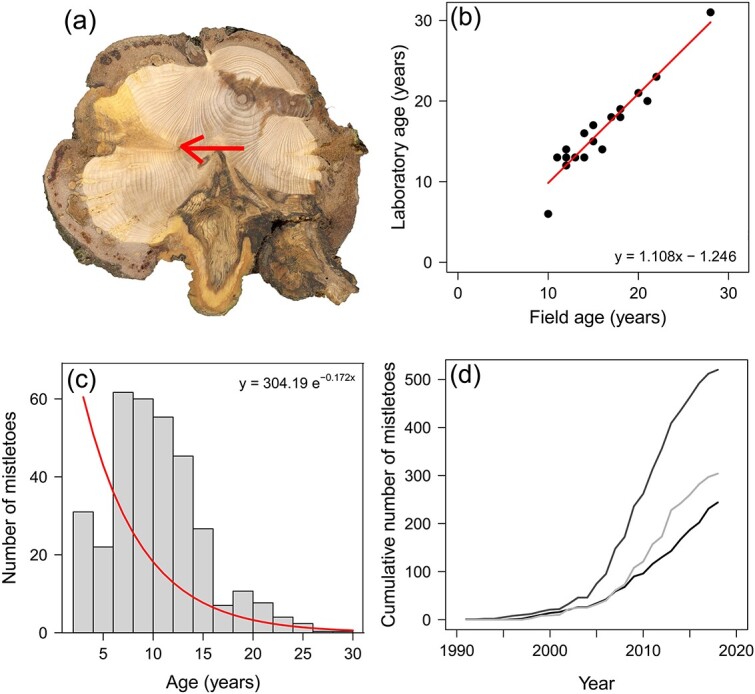
(a) Branch cross-section of silver fir infested by mistletoe. The arrow indicates the insertion of the haustorium into the tree-ring corresponding to 1994 (i.e. mistletoe age = 27 years.). (b) Estimates of mistletoe age by field and laboratory methods. The regression line and equation show the relationship between both methodologies (*R*^2^ = 0.911). (c) Frequency distribution of mistletoe age estimated in all SI fir trees. (d) Cumulative growth of the mistletoe population on each SI tree. Different lines represent different sampled trees.

In addition, we characterized biomass and age structure of mistletoe in the silver fir stand. We selected and felled three SI trees and counted the number of mistletoe individuals present in each third of the host crown. As the age of a living mistletoe directly represents the year of infection (Noetzli et al. 2003), the number of mistletoes of a certain age represents the increase in disease in the corresponding year. Age determination of mistletoes was conducted by the two methods described in (Noetzli et al. 2003). In the field, we counted shoot segments of every individual since mistletoes present regular dichotomous branching. We also collected 20 wood slices from branches or stem of felled trees at the exact point where mistletoe penetrated the bark and brought them to the laboratory. Following dendrochronological methods, we located the tip of the haustorium and counted the number of tree rings on the host branch containing the longest-lived haustorium (Fig. 1a), which corresponds to the number of years since the mistletoe became established on the branch. Field-determined and laboratory-determined age were closely related (*R*^2^ = 0.911) (Fig. 1b), so we assumed age estimation in the field is fairly accurate after correction for the regression equation. This allowed us to reconstruct mistletoe population dynamics on fir SI trees. The first year when 10 or more new mistletoe individuals were established in each tree was interpreted as the turning point between the time before the disease and a phase of exponential growth of the mistletoe subpopulations following Noetzli et al. (2003). In this study, mistletoes younger than 3 years were not included because they comprise the nonparasitic stage during the early establishment phase of mistletoe (Mathiasen et al. 2008).

### Dendrochronological methods

Two cores at 1.3-m height were extracted from each selected tree using 5-mm Pressler increment borers (Haglöf, Sweden) for dendrochronological analysis. The wood samples were air-dried, glued onto wooden mounts, and polished until the xylem cellular structure was visible (Fritts 1976). All samples were visually cross-dated, and TRW was measured with a 0.001 resolution using scanned images (resolution 2400 d.p.i.) and the CooRecorder-CDendro software (Larsson 2005). The quality of cross-dating was checked using the COFECHA software which calculates moving correlations between individual series of ring-width values and the mean sites series (Holmes 1983). Dendrochronological statistics were calculated over the best-replicated period (1950–2020) including the first-order autocorrelation (AC) and the mean sensitivity (MS) which measures relative changes in width between consecutive years. Besides, the quality and reliability of the chronologies was estimated by calculating the Expressed Population Signal (EPS) and the mean correlation among indexed ring-width series (*Rbar*) for each species at each site (Wigley et al. 1984; Briffa and Jones 1990).

The TRW series were transformed to basal area increment (BAI) series because it is a 2D measure of stem increment in area that is known to better reflect the growth of the whole tree than the 1D ring width (Biondi and Qeadan 2008). BAI series were calculated using the following Eq. (2) and assuming concentric rings:


(2) \begin{equation*} \mathrm{BAI}=\mathrm{\pi} \left({R^2}_t-{{R^2}_{t-}}_1\right), \end{equation*}

where *R^2^_t_* and *R^2^*_*t −* 1_ are the cumulative radii corresponding to the years *t* and *t* − 1, respectively.

To assess short-term growth responses to droughts, we calculated the resilience indices proposed by Lloret et al. (2011) based on the ratios of predrought, drought, and postdrought growth (BAI) values. Resistance characterizes the ability of the tree to absorb the stress situation induced by the drought event; recovery reflects the extent of growth increase or decrease after the drought event; and resilience indicates a tree’s ability to revert to predrought growth levels. We selected the three most severe drought events since 1990 based on summer SPEI, which were 1994, 2003 and 2017 at the Villanúa site and 1994 (St Clair et al. 2005) and 2012 at the Moscardón site. Pre- and postdrought periods of 3 years were considered based on previous studies (Anderegg et al. 2015; Gazol et al. 2017).

### Dendrochemistry analyses

From the above trees, we selected 10 fir trees and 8 pine trees evenly distributed between the two classes of mistletoe infestation for chemical analysis (C and O isotope composition and nutrient concentrations). Additional 10-mm-thick cores were extracted from selected fir trees, whereas material of pines was obtained from wood discs of trees cut down at the time of sampling. We separated the tree rings manually under the binocular using a scalpel and pooled them in groups of five contiguous rings at the individual tree-level (e.g. 1990–1994, 1995–1989, etc.; *n* = 6 samples per tree). We milled each group of five tree rings using a ball mill (Retsch ZM1, Haan, Germany).

Tree-ring concentration of calcium (Ca), copper (Cu), iron (Fe), potassium (K), magnesium (Mg), manganese (Mn), phosphorus (P) and sulphur (S) was measured by inductively coupled plasma optical emission spectrometry (ICP-OES; Thermo Elemental Iris Intrepid II XDL, Franklin, MA, USA) after a microwave-assisted digestion with HNO2:H2O2 (4: 1, v/v). Tree-ring nitrogen (N) concentration was measured with a combustion elemental analyser (TruSpec Micro, Leco, St Joseph, MI, USA). We also calculated three physiologically meaningful stoichiometric molar ratios N:P, N:K and P:Mn (He and Dijkstra 2014; Hevia et al. 2019).

We used whole wood instead of α-cellulose for isotopic analysis as both have been shown to provide similar environmental signals in the two study species (Barbour et al. 2001; Weigt et al. 2015). The carbon isotope composition (δ^13^C) of tree rings (5 year pool) was determined using a PDZ Europa ANCA-GSL elemental analyser interfaced to a PDZ Europa 20–20 isotope ratio mass spectrometer (Sercon Ltd, Cheshire, UK). The oxygen isotope composition (δ^18^O) was analysed using an elementar PyroCube (Elementar Analysensysteme GmbH, Hanau, Germany) interfaced to an Isoprime VisION (Isoprime Ltd, Stockport, UK, a unit of Elementar Analysensysteme GmbH, Hanau, Germany). The results were expressed relative to Vienna Pee Dee Belemnite (δ^13^C) and Vienna Standard Mean Ocean Water standards (δ^18^O). All stable isotope analyses were conducted at the Stable Isotope Facility (University of California, Davis, CA, USA).

The foliar δ^13^C was used to estimated intrinsic water-use efficiency (iWUE) as the ratio between the photosynthetic rate (*A*) and the stomatal conductance rate (*g_s_*), following Farquhar et al. (1982):


(3) \begin{equation*} \mathrm{iWUE}= Ca\times \left[1-\left( Ci/ Ca\right)\right]\times 0.625, \end{equation*}

where *Ca* and *Ci* are CO_2_ concentrations in the atmosphere and the intercellular space, respectively, and 0.625 is the relation among conductance of H_2_O and CO_2_. To determine *Ci*, we used the next equation


(4) \begin{equation*} Ci= Ca\left[\left({\mathrm{\delta}}^{13}{\mathrm{C}}_{\mathrm{tree}}-{\mathrm{\delta}}^{13}{\mathrm{C}}_{\mathrm{atm}}+1\right)/\left(b-a\right)\right], \end{equation*}

where δ^13^C_tree_ and δ^13^C_atm_ are the tree and atmospheric C isotope compositions, respectively, *a* is the diffusion fractionation across the boundary layer and the stomata (+4.4‰) and *b* is the Rubisco enzymatic biologic fractionation (+27.0‰). Estimated values of δ^13^Catm were obtained from (Belmecheri and Lavergne 2020).

### Statistical analyses

We used one-way ANOVAs to assess differences between NI and SI trees regarding C and O isotopic signals and elemental composition in tree rings. Student’s *t*-tests were employed to check if the changes through time of BAI, isotopes and nutrients differed between infestation classes in each study species. We tested the temporal changes in growth, isotopic and nutrient composition series during the common period 19,902,020 by fitting linear mixed-effects models (LMMs, Pinheiro and Bates 2000). The LMMs were fitted as follows:


(5) \begin{equation*} Y=f\left({X}_{st}\right)+{u}_s+{v}_t+{e}_{st}, \end{equation*}

where *Y* is the response variable, *f(X)* is the set of fixed effects, *u_s_* represents the tree identity random effects, *v_t_* is a normally distributed random effect for the period and *e_st_* is the normally distributed residual for tree *s* at year *t*. We used this random structure for the models following Mehtätalo et al. (2011) to study the temporal variation in growth, isotope, and nutrient composition while accounting for the fixed effects together with unspecified tree- and period-level factors. The BAI was always transformed (log[x + 1]) to fulfil normality and variance homogeneity assumptions. As fixed effects, we included year, infestation class and the interaction between them.

The impacts of soil moisture and evaporative water demand on radial growth and isotope composition of tree rings were assessed by LMMs with the random effects structure specified in Eq. (5). For each species, the response variable (BAI, iWUE, δ^18^O) was fitted against summer SPEI, VPD and their interaction with infestation class during the period 1990 to 2020. Growth models integrated tree DBH as individual level covariate. In the isotope composition models, SPEI and VPD data were averaged at 5-year intervals to match the resolution of iWUE and δ^18^O series. We also checked the covariation between tree-ring δ^18^O and monthly δ^18^O of rainfall by means of LMMs. The window of analysis of monthly δ^18^O rainfall spanned from previous September to current September of the year of tree-ring formation and the selection of the model was based on the lowest corrected Akaike information criterion (Burnham and Anderson 2002). These models were fitted separately for NI and SI trees to allow different months to impact on each infestation class. The goodness of fit of LMMs was evaluated with the coefficient of determination for GLMMs (*R_GLMM^2^*) proposed by (Nakagawa et al. 2017). Marginal *R*^2^ (*R_GLMM(m)^2^*) accounts for the proportion of variance explained by the fixed effects, and conditional *R*^2^ (*R_GLMM(c)^2^*) is the variance explained by fixed plus random effects.

To assess the nutritional status of each species and the responses to mistletoe infestation, we conducted a Principal Component Analysis (PCA) on the covariance–variance matrix considering tree-ring nutrient concentrations (N, Ca, K, S, Mg, P, Fe, Mn and Cu). We kept the first (PC1) and second (PC2) principal components because they both accounted for more than 40% of the variance. The corresponding scores of PC1 and PC2 axes of individual trees were analysed by one-way ANOVA to determine the significance of PC1 and PC2 axes to separate mistletoe infestation classes within each tree species.

We evaluated the relationship between radial growth, isotope, and nutrient composition across infestation classes considering both temporal and intertree variability within each species. To analyse associations over time, LMMs were fitted following Eq. (5) with detrended isotope and nutrient concentration as predictor variables to avoid spurious relationships that may arise from strong temporal dependence. The detrending was conducted by fitting least-squares linear regressions and subtracting the resulting function from individual tree series. To assess the relationship among individuals, we adjusted linear models using variable values averaged at the tree-level over the 21st century (i.e. period 2000 to 2020) to better capture the impact of mistletoe infestation.

All statistical analyses were conducted within the R software (R Core Team 2023). Processing of radial growth series and dendrochronological statistics were calculated using the package *dplR* (Bunn et al. 2020). Calculation of drought resilience indices was conducted with *pointRes* package (van der Maaten-Theunissen et al. 2015). The LMMs were fitted using the *lme4* and *lmerTest* packages (Bates et al. 2015; Kuznetsova et al. 2017). Estimates and confidence limits (CLs) of LMMs were calculated using the *emmeans* package (Lenth 2023). Multivariate analyses were performed with the *vegan* package (Oksanen et al. 2019).

## Results

### Mistletoe population dynamics in silver fir trees

A total of 1003 mistletoes were collected from the silver fir SI trees. The weighted average age was 11 years, and the oldest individual was 28 years old. Mistletoe age structure followed an exponential curve (*R*^2^ = 0.227) (Fig. 1c). Negative residuals for young age classes (4 to 7 years) could indicate an underestimation of these ages because they were not counted in the field. Alternatively, this result could be showing increased mortality of mistletoes during recent years due to reduced suitability of environmental conditions or declining host tree vigour. Positive residuals in the intermediate age classes (8 to 14 years) would indicate higher recruitment and/or lower mortality of these cohorts corresponding to the higher colonization rate. The mistletoe population increased since about 15 years, following a nearly exponential trend in each sampled tree (Fig. 1d). Thus, the phase of exponential growth was reached during the period 2002 to 2007 for SI firs.

### Radial growth patterns and climatic drivers

**Figure 2:**
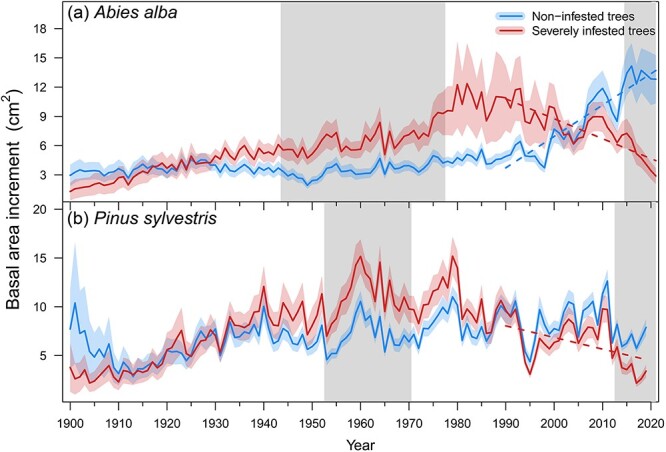
Interannual variation of BAI of noninfested and SI of silver fir (a) and Scots pine (b). Solid lines represent the means and shaded areas around them the standard error of the mean. The grey filled areas indicate periods when BAI of infestation classes significantly (*P* < 0.05) differed. Dash coloured lines represent significant trends according to linear mixed effects models (Table S1 available as supplementary data at *Tree Physiology* Online).

We found that silver fir trees of both infestation classes showed similar DBH, whereas Scots pine SI trees had a significantly higher DBH than NI trees (Table 1). No differences were found regarding tree height in either species. The canopies of SI trees were significantly more defoliated than those of NI trees in both species. During the period 1950 to 1989, NI fir trees showed narrower rings than SI fir trees, while during the period 1990 to 2020, the opposite was observed for both species (Table 1). Radial growth (BAI) significantly differed between infestation classes during the two periods in both species. During the mid-20th century, SI trees grew more than NI trees. But since 2015 (fir) and 2013 (pine), NI trees showed higher BAI values than SI trees (Fig. 2). Since 1990, NI fir trees had a positive growth trend, while SI trees of both species showed a negative trend (Table S1 available as Supplementary data at *Tree Physiology* Online).

Climatic conditions have undergone an aridification process from 1980 onward, as indicated by a steep positive trend in evaporative atmospheric water demand (VPD, both study sites) and negative trend in soil moisture (SPEI, pine site) during summer (Fig. S1b available as Supplementary data at *Tree Physiology* Online). We found significant positive response of radial growth to SPEI only in pine trees, while both species showed significant negative relationships between BAI and VPD (Table S2 available as Supplementary data at *Tree Physiology* Online). The negative effect of summer VPD was stronger on SI trees than NI trees of both species (Table 2).

**Table 2:** Estimated trends (95% lower/upper CLs) of the effects of summer climatic conditions on radial growth and tree-ring C and O isotope composition in trees from different mistletoe infestation classes. Only significant effects are shown. Different letters indicate significant differences between infestation classes.

		***Abies alba***		***Pinus sylvestris***	
		NI trees	SI trees	NI trees	SI trees
**BAI**	SPEI			0.042 (0.017/0.067)a	0.047 (0.015/0.078)a
	VPD	−0.189 (−0.267/−0.110)a	−0.244 (−0.326/−0.163)b	−0.054 (−0.054/−0.019)a	−0.121 (−0.166/−0.075)b
**iWUE**	VPD	2.480 (1.390/3.570)a	5.110 (4.020/6.200)b	3.650 (1.770/5.530)a	3.580 (1.680/5.490)a
**δ** ^**18**^ **O**	VPD	−0.198 (−0.340/−0.057)a	−0.668 (−0.866/−0.469)b	−0.361 (−0.585/−0.137)a	−0.260 (−0.484/−0.036)a

Abbreviations: intrinsic water-use efficiency (iWUE), oxygen isotope composition (δ^18^O), Standardized Precipitation Evapotranspiration Index (SPEI), vapour pressure deficit (VPD), non-infested trees (NI), severely infested trees (SI).

**Figure 3:**
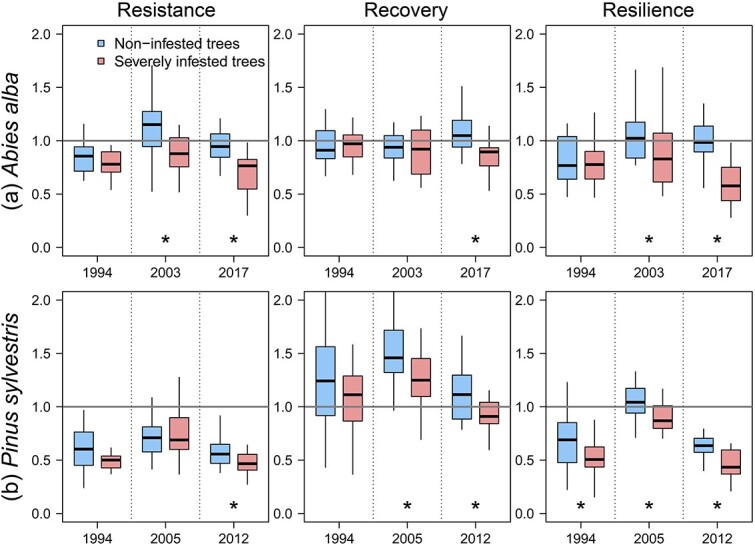
Resistance recovery and resilience indices against severe droughts during the last three decades of silver fir (a) and Scots pine (b). Significant differences (*P* < 0.05) between mistletoe infestation classes are indicated with asterisks.

Short-term growth responses to the most severe drought events recorded over the last three decades (1994, 2003 and 2017 at the Villanúa fir site and 1994 [St Clair et al. 2005] and 2012 at the Moscardón pine site) differed between NI and SI trees of both species (Fig. 3). The SI fir trees showed lower drought resistance (2003, 2017), recovery (2017) and resilience (2003, 2017) than NI fir trees (Fig. 3a). Likewise, SI pine trees had lower drought resistance (2012), recovery (2005, 2012) and resilience (1994, St Clair et al. 2005, 2012) than NI pine trees (Fig. 3b).

### Isotopic signals in tree-rings

**Figure 4:**
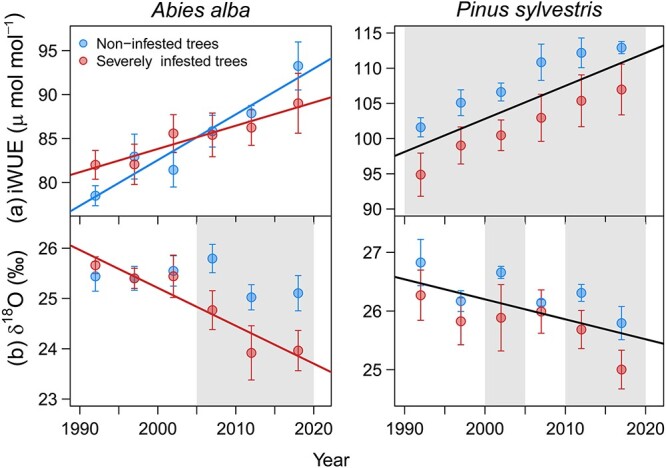
Intrinsic water-use efficiency (iWUE, a) and oxygen isotope composition (δ^18^O, b) in tree rings of different mistletoe infestation classes of silver fir and Scots pine. Symbols represent values of five consecutive rings pooled at tree level and error bars indicate standard errors among trees. The grey filled areas indicate periods when nutrient concentration significantly (*P* < 0.05) differed between infestation classes. Lines represent significant trends of each infestation class (coloured lines) or common for both classes (black lines) according to LMMs (Table S1 available as Supplementary data at *Tree Physiology* Online).

The aggregated δ^13^C throughout the 1990 to 2020 period did not significantly differ between NI and SI fir trees, but it was higher in NI than in SI pine trees (Table 3). Likewise, we did not find significant differences in iWUE between infestation classes of fir trees, but NI pine trees showed higher iWUE values than SI pine trees throughout the period 1990 to 2020. The iWUE showed an increasing trend over time in all cases, which was steeper in NI than SI trees of fir and did not differ between infestation classes in pine (Fig. 4a). Tree-ring δ^18^O was higher in NI trees than in SI trees of both species (Table 3), although these differences were not maintained throughout the period 1990 to 2020 but were significant for the last 15 years in fir and during the periods 2000 to 2005 and 2010 to 2020 in pine (Fig. 4b). Tree-ring δ^18^O showed negative trends in SI fir trees and in both infestation classes of pine trees (Fig. 4b). We found significant and positive relationship between iWUE and δ^18^O across infestation classes in fir trees, but this relationship was not significant for pine trees (Fig. S2 available as Supplementary data at *Tree Physiology* Online).

**Table 3:** Isotopic signals, nutrient concentrations and nutrient ratios in tree-rings on NI and SI trees of silver fir and Scots pine during the period 1990–2020. Different letters indicate significant differences between infestation classes according to one-way ANOVA tests.

	***Abies alba***		***Pinus sylvestris***	
	NI trees	SI trees	NI trees	SI trees
δ^13^C (‰)	−26.57 ± 0.07a	−26.56 ± 0.10a	−24.44 ± 0.09b	−25.05 ± 0.12a
iWUE (μmol mol^−1^)	84.98 ± 1.15a	85.06 ± 0.99a	108.22 ± 1.07b	101.61 ± 1.41a
δ^18^O (‰)	25.39 ± 0.12b	24.86 ± 0.19a	26.27 ± 0.29b	25.71 ± 0.42a
N (mg g^−1^)	1.45 ± 0.06a	1.51 ± 0.06a	1.95 ± 0.12a	2.34 ± 0.23a
P (μg g^−1^)	47.00 ± 4.27a	54.67 ± 5.57a	127.92 ± 15.19a	116.67 ± 12.83a
K (μg g^−1^)	530.33 ± 0.04a	701.33 ± 161.47b	756.25 ± 60.33b	531.67 ± 41.54a
Ca (mg g^−1^)	0.76 ± 0.04a	0.85 ± 0.07a	1.42 ± 0.23a	1.69 ± 0.25a
S (μg g^−1^)	99.33 ± 4.59a	112.33 ± 9.88a	167.08 ± 22.84a	178.75 ± 26.81a
Mg (μg g^−1^)	71.33 ± 5.54a	74.33 ± 5.83a	163.33 ± 14.55a	178.33 ± 17.48a
Fe (μg g^−1^)	91.93 ± 19.60a	109.38 ± 26.07a	44.51 ± 9.58a	30.48 ± 4.48a
Mn (μg g^−1^)	27.16 ± 1.33b	20.82 ± 2.47a	3.19 ± 0.63a	3.79 ± 0.32b
Cu (μg g^−1^)	2.21 ± 0.20b	1.74 ± 0.20a	1.50 ± 0.25a	1.31 ± 0.11a
N:P	87.05 ± 9.79a	75.11 ± 7.19a	40.21 ± 3.44a	51.69 ± 5.29 b
N:K	8.90 ± 0.74a	7.25 ± 0.66a	8.41 ± 1.07a	12.93 ± 1.31b
P:Mn	3.30 ± 0.38a	5.78 ± 0.59b	118.39 ± 19.22b	86.50 ± 32.47a

Abbreviations: carbon isotope composition (δ^13^C), intrinsic water-use efficiency (iWUE) oxygen isotope composition (δ^18^O), and concentrations of nitrogen (N), calcium (Ca), potassium (K), sulphur (S), magnesium (Mg), phosphorus (P), iron (Fe), manganese (Mn) and copper (Cu).

We only found significant responses of tree-ring isotope composition to variations in evaporative water demand (VPD) but not to soil water shortage represented by SPEI (Table S2 available as Supplementary data at *Tree Physiology* Online). The iWUE of both species showed positive relationships with VPD, which was stronger for SI trees than for NI trees of fir but similar between infestation classes of pine (Table 2). Tree-ring δ^18^O of both species negatively responded to increases in VPD, although the effect was stronger in SI than NI fir trees (Table 2). Moreover, tree-ring δ^18^O was positively correlated with rainfall δ^18^O composition. Both infestation classes of pine showed the strongest association with rainfall δ^18^O values during April, whereas NI fir trees responded to rainfall δ^18^O values during April and SI trees to rainfall δ^18^O values during February (Fig. S3 available as Supplementary data at *Tree Physiology* Online).

### Elemental composition of wood

We found different patterns between species in their tree-ring wood elemental composition (Table 3). In fir trees, Cu and Mn concentrations were higher in NI trees than in SI trees, while the opposite pattern was found regarding K concentration. In pine trees, NI trees showed higher concentration of K and lower concentration of Mn than SI trees (Table 3). The P:Mn stochiometric ratio was higher in SI fir trees than in NI fir trees, and in pine SI trees, it showed higher N:P and N:K and lower P:Mn ratios than NI trees (Table 3). The PCA analyses of wood multi-elemental composition revealed neither significant differences between tree classes with respect to scores along PC1 neither in firs (ANOVA; *F* = 0.051, *P* = 0.822) nor in pines (*F* = 0.553, *P* = 0.461) (Fig. S4 available as Supplementary data at *Tree Physiology* Online). With respect to scores along PC2, fir trees were similar (*F* = 0.498, *P* = 0.483), but we found differences in pine trees (*F* = 4.477, *P* = 0.040), with SI trees showing higher scores than NI trees. This PC2 axis was mainly related positively to N concentration and negatively to K and P concentrations in wood.

**Figure 5:**
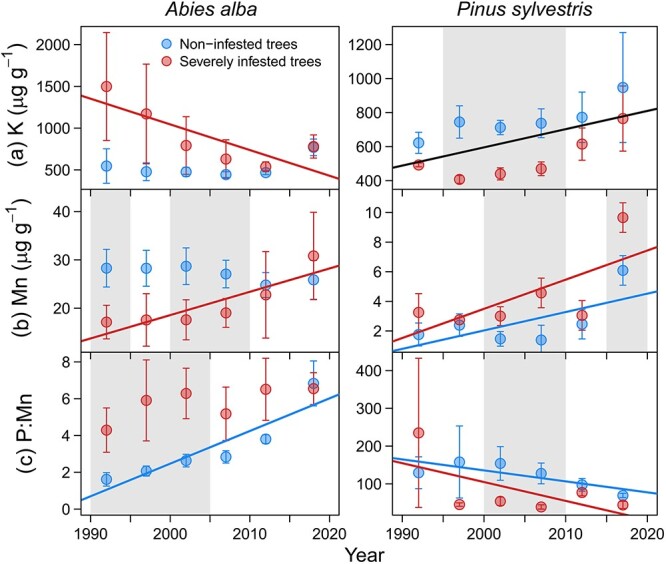
Temporal variation in tree-ring nutrient concentrations in trees of different mistletoe infestation classes of silver fir and Scots pine. Symbols represent the means and bars are standard errors of each group of five consecutive rings. The grey filled areas indicate periods when nutrient concentration significantly (*P* < 0.05) differed between infestation classes. Lines represent significant trends of each infestation class (coloured lines) or common for both classes (black lines) according to LMMs (Table S1 available as Supplementary data at *Tree Physiology* Online).

Over the period 1990 to 2020, tree ring nutrient concentrations showed significant temporal trends (Table S1 available as Supplementary data at *Tree Physiology* Online). The most notable results include reductions in K concentration over time in SI fir trees and increases in pine trees without differences between infestation classes (Fig. 5a). The Mn concentration increased over time in SI trees significantly and/or more pronounced than in NI trees of both species (Fig. 5b). We also found P:Mn imbalances related to mistletoe infestation. Firs and pines showed positive and negative trends, respectively, but SI trees of both species have experienced greater reductions in P content relative to Mn than NI trees over time (Fig. 5c).

### Relations of carbon, water and nutrient balances and tree growth

**Figure 6:**
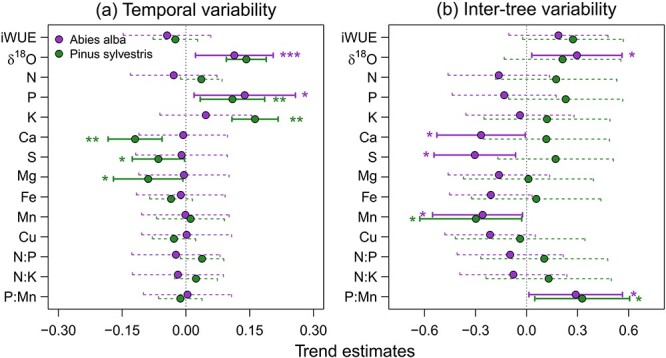
Relationships between BAI and isotope and nutrient composition in tree rings through time (a) and across individual trees (b). Symbols represent estimated standardized effect and error bars 95% CLs. Significant and nonsignificant effects are represented by solid and dashed lines, respectively. Associated probability (^*^P < 0.05; ^*^^*^P < 0.01; ^*^^*^^*^P < 0.001) of each variable is shown.

We found significant relationships between radial growth and isotope and nutrient composition across infestation classes in both species. Considering temporal variability, radial growth was positively related to δ^18^O and P concentration in both species. Pine growth over time was positively linked to K concentration and negatively linked to Ca, S and Mg concentration in wood (Fig. 6a) Regarding variability among individuals, we found that trees with lower Mn concentrations and higher P:Mn ratios in wood showed higher radial growth in both conifer species. Fir trees with higher δ^18^O and lower Ca and S concentration in their wood also had higher growth rates (Fig. 6b).

## Discussion

### Impact of mistletoe infestation on tree growth and water-use efficiency

The infestation by mistletoe (*V. album*) modified radial growth patterns of the studied fir and pine trees, evidenced by declining growth and significantly lower growth rates of SI trees than NI trees. Indeed, during the mid-20th century, SI trees were growing more vigorously, likely related to the main mechanism of mistletoe dispersal by avian vectors. Bird species prefer perching on bigger and taller trees, so the most dominant trees in the forest canopy are the most likely to become infested (Durand-Gillmann et al. 2014; Sangüesa-Barreda et al. 2018). The water and nutritional status of host trees has also been correlated with mistletoe growth, so healthier trees with better past growth would also harbour greater mistletoe biomass (Shaw et al. 2005; Rigling et al. 2010). Negative temporal growth trends of SI trees over the last 30 years, along with the loss of resilience against successive droughts, hint at a cumulative detrimental impact of mistletoe on tree performance. Likely, this fact is linked to the onset in the mistletoe population exponential growth (Noetzli et al. 2003), at least in the case of silver fir. Thus, mistletoe infestation completely reversed the previous growth trends in forest stands by differentially impairing the growth of large-dominant individuals. Many previous studies have reported consistent negative effects of mistletoe on host growth in both silver fir (Noetzli et al. 2003; Durand-Gillmann et al. 2014) and Scots pine (Sangüesa-Barreda et al. 2013; Yan et al. 2016; Bilgili et al. 2018). Decreased whole-canopy leaf area and increased tree crown transparency are considered major factors driving growth decline and mortality in mistletoe-infested trees and stands (Dobbertin and Rigling 2006; Kollas et al. 2018). The reduction in photosynthetically active crown leaf area has been associated with needle loss due to hydraulic occlusions caused by mistletoe sinkers and haustorium intrusion into xylem (Meinzer et al. 2004; Xia et al. 2012), and with morphological and physiological modifications of needles, such as decreased needle size or chlorophyll content (Reblin et al. 2006; Rigling et al. 2010; Ozturk et al. 2022).

Mistletoe is considered as both a predisposing factor for tree death by increasing needle loss and a contributing factor by amplifying drought stress (Tsopelas et al. 2004; Dobbertin and Rigling 2006; Sangüesa-Barreda et al. 2012). The greater climatic sensitivity of SI trees than their healthy counterparts of both species (Table 2) support our first hypothesis. It is challenging to distinguish whether low soil moisture availability or elevated atmospheric water demand further hinders tree growth (Gazol and Camarero 2022), but the differential growth response to VPD between infestation classes supports the prevalent role of air temperature and atmospheric dryness in the responses of mistletoe-infested stands (Grossiord et al. 2020; Griebel et al. 2022). We also found a deterioration of growth response to droughts of SI trees, an early-warning sign of tree mortality (DeSoto et al. 2020), as indicated by the significant differences between infestation classes in resilience indices during recent droughts that were not observed in the 1994 drought. Therefore, the direct effects of mistletoe on tree water status, together with the indirect effects (i.e. reduced canopy leaf area), likely increase drought stress experienced by SI trees and hinder their recovery, so that the negative impact of unfavourable climatic conditions on radial growth may be amplified. In fact, the combination of both factors has been associated with increases in fir and pine mortality in the study regions (Oliva and Colinas 2010; Sangüesa-Barreda et al. 2013).

Concurrent with growth declines in SI trees and aligned with previous research (Sangüesa-Barreda et al. 2013; Marias et al. 2014), iWUE patterns differed between mistletoe infestation classes. Our results are in accordance with foliar iWUE observations (Sala et al. 2001; Meinzer et al. 2004; Logan et al. 2013), thus providing evidence that fractionation events that influence δ^13^C at the leaf level are imprinted also on tree-rings (Gessler et al. 2014). We found smaller iWUE increases over time (fir) and systematically lower iWUE (pine) in SI trees compared with NI trees, suggesting either diminished *A* or enhanced *g_s_* or a combination of both (Farquhar et al. 1989). Reduced carbon uptake capacity has received greater support as driver of impaired iWUE in mistletoe-infested trees, as indicated by the lower concentration of nonstructural carbohydrates in infested trees (Meinzer et al. 2004; Sangüesa-Barreda et al. 2012; Yan et al. 2016). Several mechanisms have been proposed to drive such impairment of carbon assimilation, including reductions of canopy leaf area and sapwood (Galiano et al. 2011; Kollas et al. 2018), or decreased nitrogen content of needles (Meinzer et al. 2004; Mutlu et al. 2016b). Nevertheless, there is little evidence that mistletoe actively extracts carbohydrates from its hosts (Galiano et al. 2011; Wang et al. 2022; but see also Escher et al. 2004).

### Interpretation of tree-ring δ^18^O response to mistletoe infestation

The δ^18^O composition of tree tissues is primarily influenced by variations in the isotopic signature of tree water sources, and changes in leaf evaporative enrichment determined by environmental conditions (Gessler et al. 2009; Roden and Siegwolf 2013). We disregard differences in ambient relative humidity, since SI and NI trees were growing together in the same stands and were of similar size. Fir and pine trees showed differences in the relationship between the C and O isotope composition of tree rings. The positive relationship between iWUE and δ^18^O across infestation classes found in fir trees implies that carbon assimilation was primarily governed by stomatal control of water losses (Roden and Farquhar 2012; Billings et al. 2016). Thus, the dual-isotope approach predicts that the lower δ^18^O of SI fir trees indicates higher *g_s_* of heavily infested trees of this species, considering the inverse relationship between *g_s_* and transpirative isotopic enrichment of leaf lamina water, and hence cellulose δ^18^O (Scheidegger et al. 2000; Grams et al. 2007; Siegwolf et al. 2023). This interpretation contrasts with previous field-based measurements of *g_s_* in mistletoe-infested trees that reported negative associations between *g_s_* and mistletoe infestation levels (Zweifel et al. 2012; Kubov et al. 2020; Griebel et al. 2022, although see Scalon et al. 2021). Host tree stomatal closure has been proposed as a mechanism to prevent leaf turgor loss or stem hydraulic failure when mistletoe and host tree compete for limiting water (Glatzel and Geils 2009; Wang et al. 2022). However, the lower ratio of leaf area to sapwood area of SI fir trees due to their severe defoliation may favour tree transpiration flux concentration into fewer leaves and thus increase sap flow rates per individual leaf and, therefore, increase *g_s_* and transpiration rates per unit leaf area (Mencuccini and Grace 1995). Conversely, the lack of significant associations between carbon and oxygen isotopic signals in pine trees revealed an uncoupling between tree-ring δ^18^O and stomatal responses to environmental conditions (Barbour et al. 2000).

The negative relationships between δ^18^O and VPD found in both tree species is contrary to the expected *g_s_* reduction under high evaporative demand (Grossiord et al. 2020), which together with the uncoupling between leaf-level processes and oxygen isotope signature (pine) provide evidence that stomatal regulation was not the only driver of tree-ring δ^18^O variability. It is important to note that tree-ring δ^18^O also reflects the imprint of the isotopic signal of the source water used by the tree (Sarris et al. 2013; Wang et al. 2021). A plastic behaviour of tree species capable of shifting water uptake from dry topsoil to deeper, moister soil layers, subsoil or weathered bedrock during drought is increasingly recognized (Treydte et al. 2014; Voltas et al. 2015), and has been proposed to explain differences in drought-induced canopy dieback and tree mortality processes (Ripullone et al. 2020; González de Andrés et al. 2021, 2022). In our study, the lower tree-ring δ^18^O values of SI trees hint at a shift in water uptake depth as a consequence of enhanced mistletoe-induced drought stress, which forces trees to use a greater proportion of ^18^O-depleted water from deep soil layers replenished by winter rainfall and snowmelt (Sarris et al. 2013; Siegwolf et al. 2023). This interpretation is further supported for fir, since the tree ring δ^18^O values of SI trees are aligned with the isotopic signature of winter precipitation, in contrast to NI trees in which tree ring δ^18^O values are aligned with the isotopic signature of spring precipitation.

Yet, another alternative or complementary interpretation of tree-ring δ^18^O patterns lies in a temporal mismatch in the organic matter deposition in wood cellulose between infestation classes, since isotopic signals are mainly recorded in tree-ring wood during periods of high photosynthesis and C assimilation rates (Gessler et al. 2014). During the hot, dry summers typical of the Mediterranean climate, the water deficit constrains cambial activity (Körner 2015), resulting in no or negligible tree radial growth during this period (Camarero et al. 2010). Thus, what is actually imprinted in the tree rings may be the isotopic signal of cooler and wetter spring and early summer conditions, with lower values of δ^18^O precipitation (Sarris et al. 2013; Pflug et al. 2015). Increased mistletoe-induced water stress may have arrested cambial activity earlier in SI trees, which might account for SI trees capturing less-δ^18^O enriched water signal and undergoing less evaporative enrichment of the leaf water compared with NI trees (Siegwolf et al. 2023). Consistently, we found that years in which tree-ring δ^18^O values were higher corresponded to years of higher growth in our two study species, as well as fir trees with higher δ^18^O values showing higher growth rates. The earlier association between rainfall δ^18^O and tree-ring δ^18^O in SI (rainfall δ^18^O values during February) than NI trees (rainfall δ^18^O values during April) of fir is also in accordance with this interpretation. Moreover, phenological differences also concur with lower iWUE caused by a higher *g_s_* during cooler and wetter spring conditions in SI trees, while presumably the extended growing season of NI trees during periods of more severe water shortage would favour a higher iWUE.

Previous research has also proposed changes in the transpiration-driven Péclet effect (Marias et al. 2014), or the exchange of organic oxygen atoms with unenriched xylem water during cellulose synthesis (Gessler et al. 2009) as explanations of variation of tree-ring δ^18^O under mistletoe infestation. In addition, our results might be biased because we measured δ^18^O in whole wood rather than α-cellulose (Helle et al. 2022). However, we do not consider this to be a major shortcoming, since strong correlations between the δ^18^O signatures of whole wood and cellulose and very similar responses to environmental factors in both of them have been reported, particularly for conifers including our target species (Weigt et al. 2015; Barbour et al. 2021; Ren et al. 2023). All in all, we conclude that tree-ring δ^18^O measured in wood is an information-rich and complex signal that is affected by leaf-level evaporative effects, source water isotopic signal, and/or changes in growth timing and phenology, among others. All these complex effects could be superimposed on tree ring δ^18^O, perhaps operating simultaneously, and all the interpretations outlined above are equally plausible and certainly nonmutually exclusive.

### Mistletoe-induced changes in wood elemental composition

Plant stoichiometry is largely determined by species identity (Sardans et al. 2015) and soil nutrient availability (Chen and Chen 2021), yet other processes can alter elemental composition of trees under severe mistletoe infestation. The passive pathway generated by high *g_s_* and transpiration rates allows mistletoe to capture water and nutrients from xylem sap of the host tree (Glatzel and Geils 2009; Muche et al. 2022). Consequently, mistletoe-infested trees may suffer nutrient deficiencies as suggested by lower leaf nutrient concentrations than NI trees (Ehleringer et al. 1985; Hosseini et al. 2007; Mutlu et al. 2016*b*; Scalon et al. 2021). Differences in tree-ring nutrient patterns between SI trees and their healthy counterparts can provide insights into the impact of mistletoe infestation on tree nutritional imbalances. On one hand, our results indicate that SI trees may be K-deficient as shown by the negative temporal trends of K concentration in SI firs and the chronically lower K concentration in SI pines. Potassium is involved in key physiological processes of survival and drought tolerance, such as stomatal control, hydraulic conductivity, osmoregulation and photosynthesis (da Silva et al. 2011; Trifilò et al. 2011; Sardans and Peñuelas 2015). In fact, those periods when pine trees had higher K concentrations in tree-ring wood coincided with the periods when trees grew more. Hence, we suggest that impaired K nutrition in SI trees could exacerbate and aggravate the combined stress induced simultaneously by drought and mistletoe infestation.

Phosphorus has also important functions in carbon assimilation, protein synthesis and water-use efficiency (da Silva et al. 2011; Gessler et al. 2017), which is supported by the positive association in the temporal variability between P concentration and growth. We found no evidence of P depletion in tree wood induced by mistletoe infestation, which may be driven by the soil P-mobilizing strategy deployed by plants under conditions of nutrient and drought stress through the exudation of carboxylates by roots (Lambers et al. 2013). However, this mechanism has an elevated carbon cost and often leads to an increase of Mn concentration in plant tissues (Lambers et al. 2015). Consistently, we found a greater increase over time of Mn concentration, along with imbalances of P:Mn ratios, in SI trees of both species. High Mn accumulation poses negative impacts on tree functioning since it is associated with impaired photosynthesis and growth, due to metabolic interferences with other nutrients (St Clair et al. 2005). Indeed, our results indicate that trees with higher Mn concentrations and lower P:Mn ratios in wood have worse growth performance. This study contributes to the growing body of knowledge that identifies imbalances in K and Mn nutrition as early-warning signals of loss of tree vitality and impending dieback (Houle et al. 2007; Hevia et al. 2019; González de Andrés et al. 2022; He et al. 2024).

The observed changes in nutrient concentrations and stoichiometric ratios in response to mistletoe infestation are consistent with global patterns reported in drought-induced forest dieback (He and Dijkstra 2014; He et al. 2024), which have been also observed in firs and pines (Salazar-Tortosa et al. 2018; He et al. 2019; Hevia et al. 2019; González de Andrés et al. 2022). Indeed, Lázaro-González et al. (2021) found that the metabolic profile of mistletoe-infested *Pinus nigra* trees largely coincided with that of drought-stressed trees. Hence, our results only partially validate our third hypothesis, as mistletoe could indirectly affect tree nutrition due to alterations of water-use strategies. Firstly, reductions in whole-tree transpiration resulting from mistletoe-induced canopy defoliation can disrupt transpiration-driven mass flow of nutrients from soil to roots, as suggested by the frequent negative associations between δ^18^O and nutrient concentrations (Gessler et al. 2017; Salazar-Tortosa et al. 2018). Moreover, impaired K nutrition may reduce xylem hydraulic conductivity (Nardini et al. 2011; Oddo et al. 2014), which may further contribute to impair the hydraulic functioning of the trees through a detrimental feedback mechanism. Moreover, the positive δ^18^O–growth relationship found across infestation classes would be consistent with greater water uptake from deeper soil/bedrock layers by SI trees where nutrient availability is lower than in topsoil horizons (Jobbágy and Jackson 2001; Querejeta et al. 2021). Both explanations are plausible and nonmutually exclusive, so these complex effects could occur simultaneously. Future research evaluating the mechanisms driving the carbon–water balance and nutritional impairment of severely mistletoe-infested trees in seasonally dry regions could benefit from the assessment of seasonal leaf transpiration dynamics and isotopic signatures of xylem and soil water, as proxies of tree water uptake depth (Dawson et al. 2002; Ding et al. 2021).

## Conclusions

The combined analysis of TRW, C and O stable isotope composition and nutrient concentrations are useful means to decipher the physiological processes that trigger the loss of vitality induced by mistletoe infestation. The higher sensitivity of radial growth of severely mistletoe-infested trees to high VPD is consistent with the poorer increase in iWUE of SI silver fir trees and systematically lower iWUE of SI Scots pine trees during the last three decades. The lower tree-ring δ^18^O signature of mistletoe-infested trees can be the result of increases in *g_s_* per unit leaf area driven by lower total canopy leaf area to sapwood ratios leading to transpiration flow concentration into fewer leaves, greater uptake of isotopically depleted water from deeper soil and subsoil layers, and/or different seasonal timing of organic matter deposition in wood. All these potential mechanisms are plausible and nonmutually exclusive and may have superimposed effects on tree ring δ^18^O. Tree-ring nutrient patterns do not distinctly depict nutrient impairment due to direct uptake by mistletoe but instead suggest an indirect effect of mistletoe-induced aggravation of drought stress, as indicated by K nutrient deterioration and Mn accumulation in mistletoe-infested trees. Feedbacks between carbon–water balance and tree nutrition can contribute to explain the radial growth decline of silver fir and Scots pine trees subjected to the concurrent impact of mistletoe infestation and increasing atmospheric dryness and water demand. Our findings have important implications since both mistletoe infestation and drought stress have been associated with increased mortality in rear-edge populations forming the xeric distribution limit of these conifer species (Tsopelas et al. 2004; Oliva and Colinas 2010; Sangüesa-Barreda et al. 2013).

## Supplementary Material

Supplementary_data_R1_tpae106

## Data Availability

The data supporting the results of this study are publicly available at the (to be established after acceptance).
