# Supplementary material for: Mistletoe-induced carbon, water and nutrient imbalances are imprinted on tree rings
Source: Tree Physiol. 2024 Aug 20;44(9):tpae106. doi: 10.1093/treephys/tpae106 (PMC11404520; doi:10.1093/treephys/tpae106)
Supplement: Supplementary_data_R1_tpae106 [file supplementary_data_r1_tpae106.docx]

**Supporting data for the article:**

**Mistletoe-induced carbon, water and nutrient imbalances are imprinted on tree rings**

Ester González de Andrés, Antonio Gazol, José Ignacio Querejeta, Michele Colangelo, J. Julio Camarero

**TABLES**

**Table S1.** Trends of growth, isotopic signals and nutrient composition of tree rings during the period 1990-2020.

**Table S2.** Linear mixed-effects models characterizing relationships of drought index (SPEI) and vapour pressure deficit (VPD) with radial growth and stable isotope composition.

**FIGURES**

**Figure S1.** Climate diagrams and interannual variability of summer soil moisture and atmospheric water demand at the study sites.

**Figure S2.** Relationships between intrinsic water-use efficiency and oxygen isotope composition in tree rings.

**Figure S3.** Relationships of tree-ring and rainfall oxygen isotope composition.

**Figure S4.** Principal component analysis of tree-ring nutrient concentration.

**Table S1.** Linear mixed-effects models characterizing temporal trends of growth, isotope signals and nutrient composition of tree rings for the study species silver fir (*Albies alba*) and Scots pine (*Pinus sylvestris*). Considered fixed effects are calendar year (Year), mistletoe infestation class (Class) and the interaction between them. For each variable, the *F* statistic and the associated probability (+*p* < 0.1; **p* < 0.05; ***p* < 0.01; ****p* < 0.001) are shown.

|  | ***Abies alba*** | | | ***Pinus sylvestris*** | | |
| --- | --- | --- | --- | --- | --- | --- |
|  | Year | Class | Year x Class | Year | Class | Year x Class |
| BAI ^a^ | 1.48 | 368.22*** | 369.73*** | 4.80* | 88.12*** | 89.33*** |
| iWUE | 87.79*** | 9.33** | 9.33** | 88.90*** | 0.01 | 0.01 |
| δ^18^O | 14.28* | 24.04*** | 24.25*** | 4.73* | 0.34 | 0.32 |
| N | 6.49* | 1.14 | 1.14 | 2.40 | 0.02 | 0.02 |
| P | 16.05* | 0.04 | 0.04 | 9.12* | 0.30 | 0.30 |
| K | 2.26 | 6.31* | 6.25* | 8.19** | 0.05 | 0.05 |
| Ca | 3.49^+^ | 6.69* | 6.72* | 2.69 | 1.01 | 1.02 |
| S | 18.00* | 3.59^+^ | 3.62^+^ | 5.35 | 3.75^+^ | 3.76^+^ |
| Mg | 0.11 | 2.75 | 2.74 | 2.48 | 3.59^+^ | 3.61^+^ |
| Fe | 2.39 | 1.32 | 1.33 | 1.17 | 0.30 | 0.30 |
| Mn | 2.66 | 8.31** | 8.22** | 3.74* | 7.80** | 7.77** |
| Cu | 6.33* | 0.16 | 0.15 | 6.07* | 0.48 | 0.49 |
| N:P | 27.69*** | 3.27 | 3.25 | 14.32 | 0.95 | 0.93 |
| N:K | 0.05 | 7.41** | 7.36** | 0.04 | 1.69 | 1.65 |
| P:Mn | 17.77** | 3.78* | 3.70* | 3.91* | 0.02 | 4.02* |

Abbreviations: basal area increment (BAI), intrinsic water-use efficiency (iWUE) oxygen isotope composition (δ^18^O), concentrations of nitrogen (N), calcium (Ca), potassium (K), sulphur (S), magnesium (Mg), phosphorus (P), iron (Fe), manganese (Mn), and copper (Cu).

^a^ Note that BAI has an annual resolution and the other variables were calculated in groups of five-year tree-rings.

**Table S2.** Selected linear mixed-effects models characterizing the relationship of summer soil moisture and evaporative water demand with radial growth and carbon and oxygen isotope signatures in tree rings of silver fir (*Abies alba*) and Scots pine (*Pinus sylvestris*) across mistletoe infestation classes. For each variable, the *F* statistic and the associated probability (^+^*p* < 0.1; **p* < 0.05; ***p* < 0.01; ****p* < 0.001) are shown. Note that resolution of BAI and iWUE - δ^18^O is annual and 5-year, respectively.

| **Species** | **Variable** | **BAI** | **iWUE** | **δ^18^O** |
| --- | --- | --- | --- | --- |
| Silver fir (*Abies alba*) | DBH | 8.087* | – | – |
|  | Class | 0.118 | 0.011 | 1.812 |
|  | SPEI | 1.004 | 0.068 | 2.955 |
|  | VPD | 7.928** | 97.704*** | 33.786*** |
|  | Class x SPEI | 2.529 | 3.220 | 0.459 |
|  | Class x VPD | 56.078*** | 11.745** | 14.291*** |
|  | *R_GLMM(m)^2^* | 0.217 | 0.377 | 0.320 |
|  | *R_GLMM(c)^2^* | 0.558 | 0.804 | 0.731 |
| Scots pine (*Pinus sylvestris*) | DBH | 29.935*** | – | – |
|  | Class | 4.674* | 4.159^+^ | 1.862 |
|  | SPEI | 10.873** | 3.114 | 3.687^+^ |
|  | VPD | 20.549*** | 31.067*** | 15.801*** |
|  | Class x SPEI | 0.045 | 0.004 | 0.007 |
|  | Class x VPD | 12.776*** | <0.001 | 0.416 |
|  | *R_GLMM(m)^2^* | 0.263 | 0.415 | 0.267 |
|  | *R_GLMM(c)^2^* | 0.595 | 0.693 | 0.659 |

Abbreviations: diameter at breast height (DBH), mistletoe infestation class (Class), basal area increment (BAI), intrinsic water-use efficiency (iWUE), oxygen isotope composition (δ^18^O), standardized precipitation- evapotranspiration index (SPEI), and vapour pressure deficit (VPD).


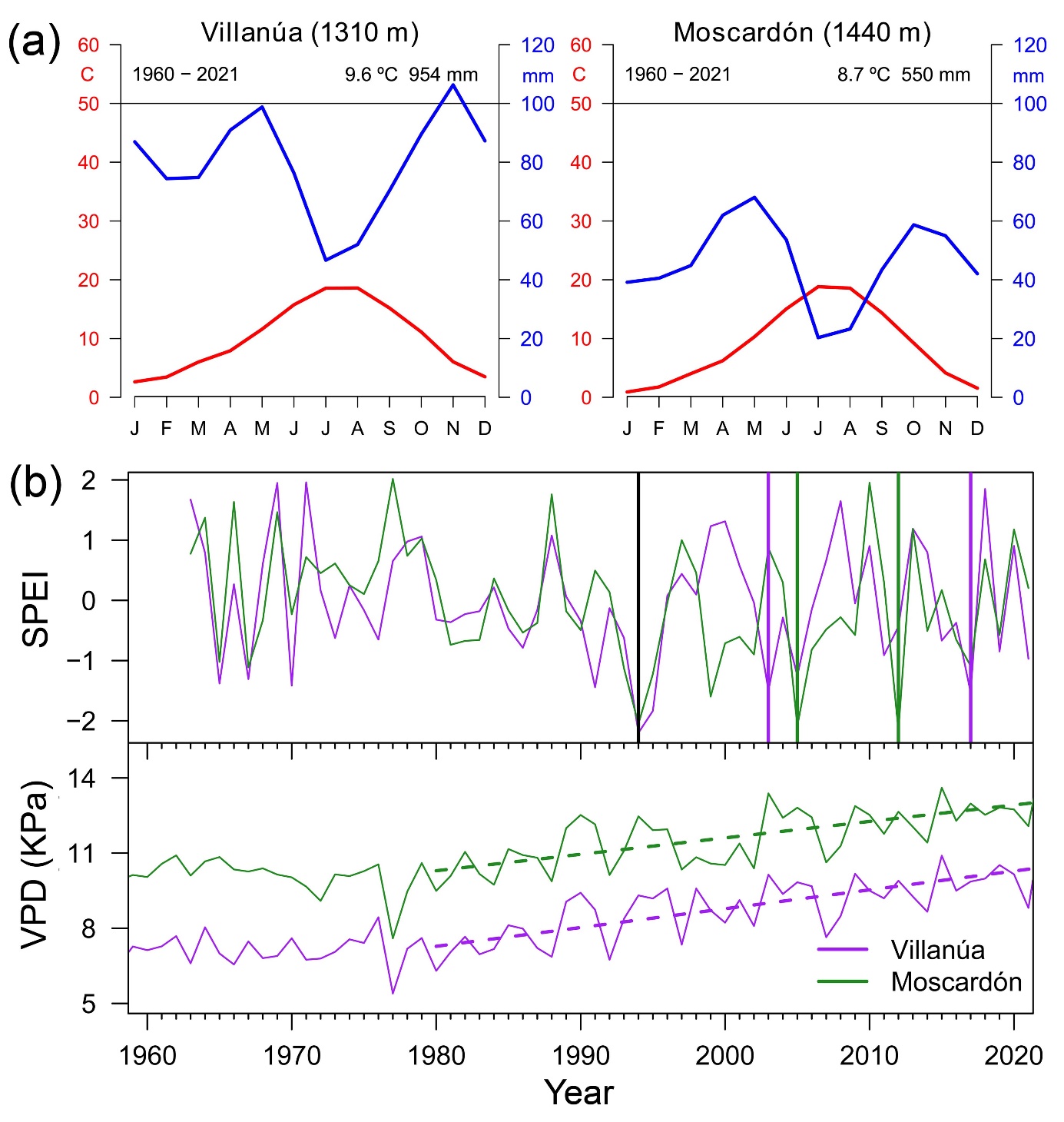


**Figure S1.** (a) Climatic diagrams for each study site according to climatic data retrieved from E- OBS v. 26.0e database (Cornes et al., 2018) for the period 1980 – 2021. (b) Standardized Precipitation Evapotranspiration Index (SPEI) and vapor pressure deficit (VPD) during summer (JJA) variability during the period 1960-2020 in the two study sites. Vertical solid lines indicate selected years of severe droughts (upper panel) and dashed lines represent significant temporal trends according to linear models (bottom panel).

**
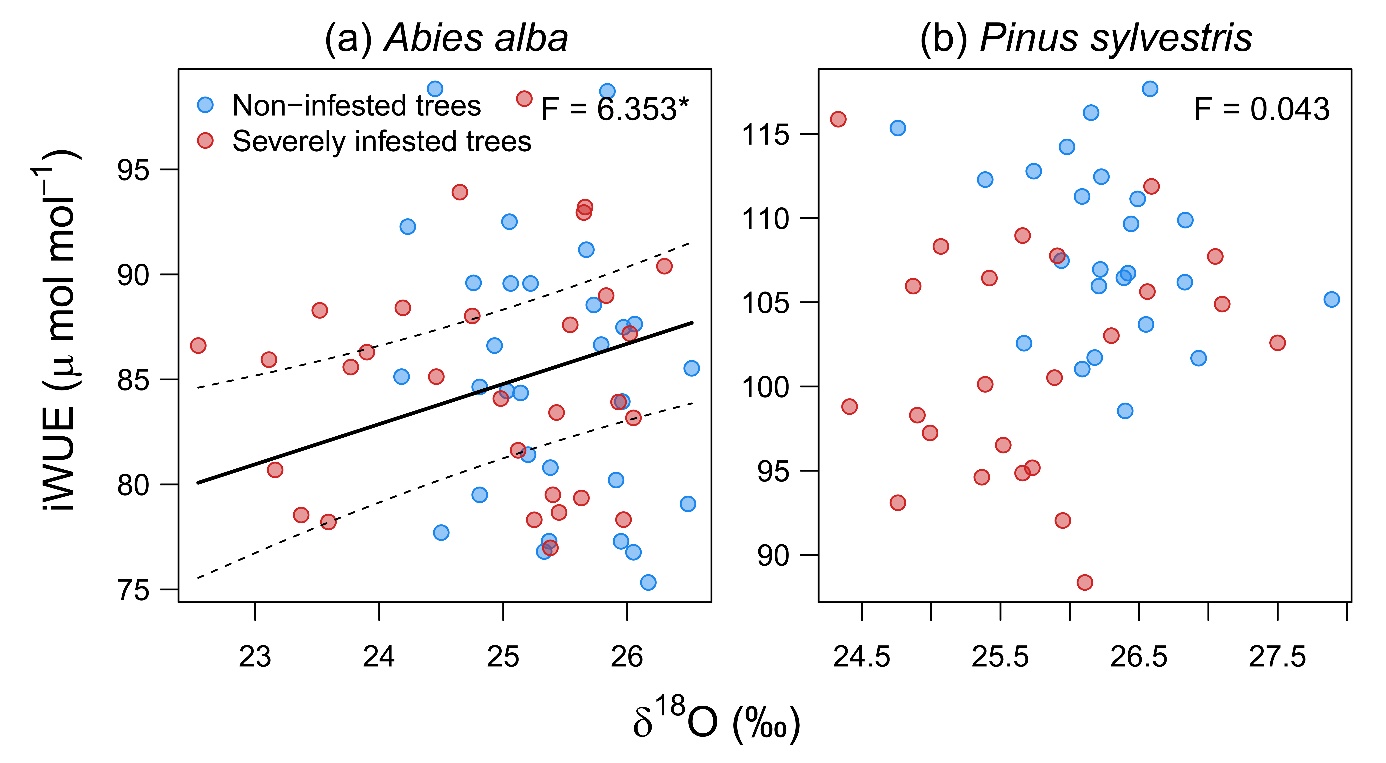
**

**Figure S2.** Relationships between intrinsic water-use efficiency (iWUE) and oxygen isotope composition (δ^18^O). Symbols represent values of five consecutive rings. Solid lines represent significant associations between isotopic signatures according to linear mixed effects models and dashed lines are 95% confidence intervals. The *F* statistic with its associated probability (**p* < 0.05; ***p* < 0.01; ****p* < 0.001) of the linear mixed-effects models are shown.


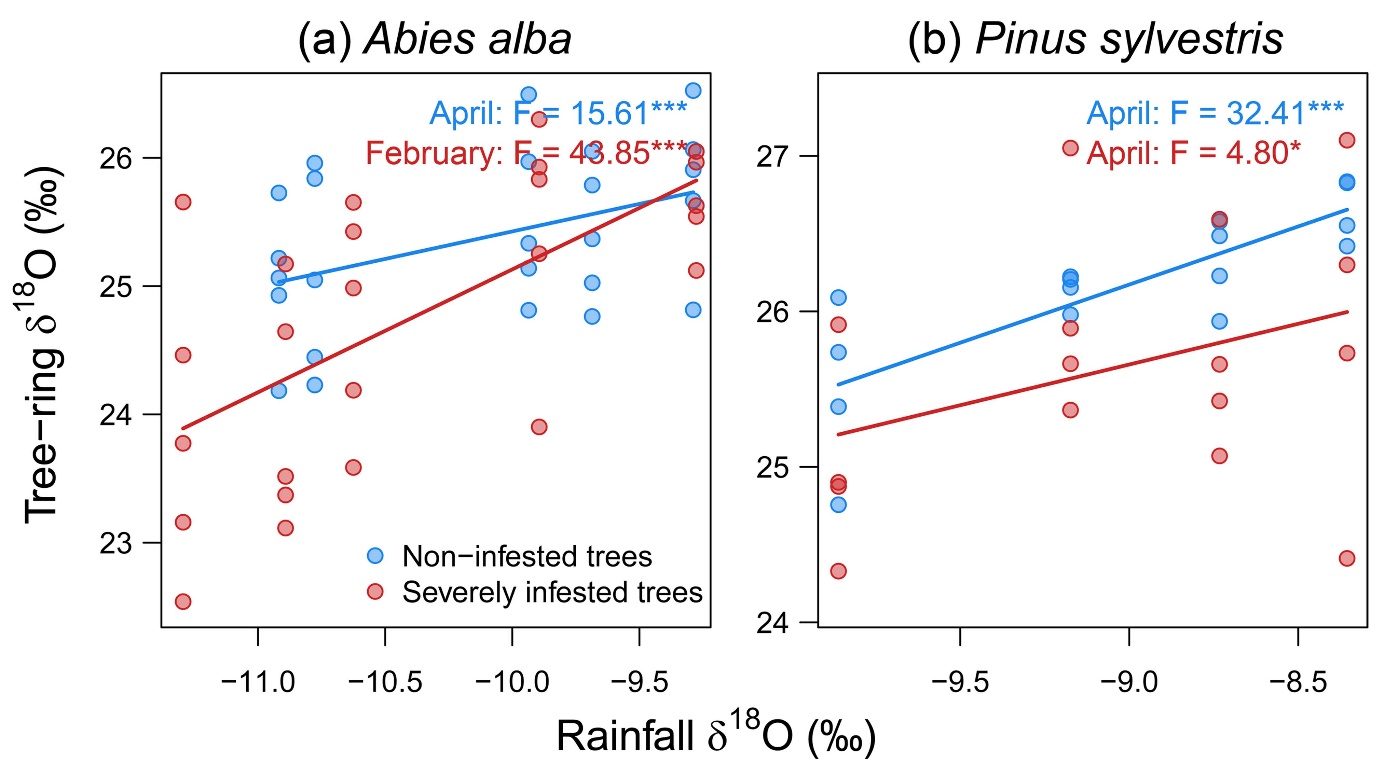


**Figure S3.** Relationships of tree-ring and rainfall oxygen isotopic composition (δ^18^O) of silver fir (a) and Scots pine (b). Symbols represent values of five consecutive rings. Solid lines represent significant associations between isotopic signatures according to linear mixed effects models. The *F* statistic and significance levels (**p* < 0.05; ***p* < 0.01; ****p* < 0.001) for the selected months of rainfall δ^18^O are shown for each mistletoe infestation class.

**
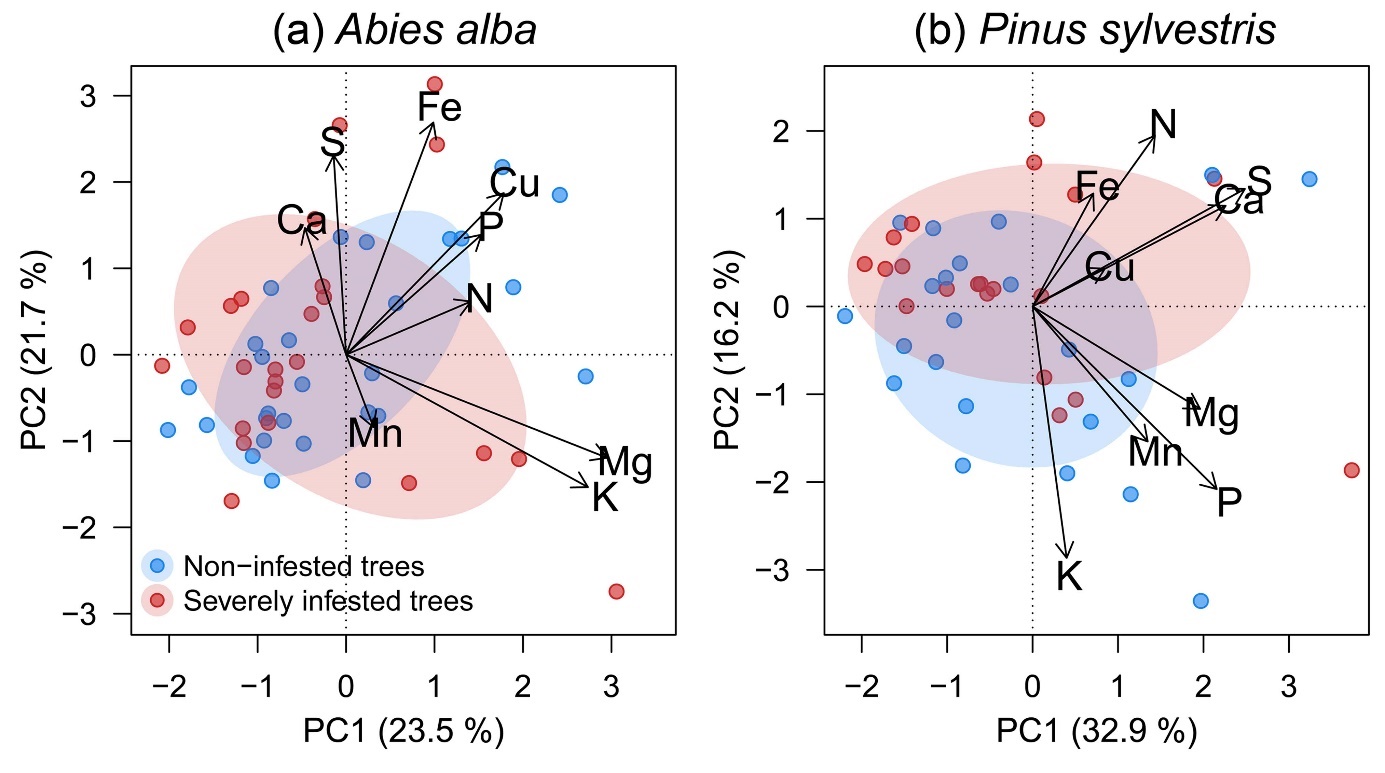
**

**Figure S4.** Principal component analysis (PCA) biplot of nutrient concentrations in tree rings of silver fir (a) and Scots pine (b). Symbols represent values of five consecutive rings and shaded areas are the centroid of the values of non-infested tress (blue) and severely infested trees (red) by mistletoe calculated at 95% probability. Black arrows indicate loadings of nutrient concentrations.
